# Hierarchical Metal–Organic Frameworks with Macroporosity: Synthesis, Achievements, and Challenges

**DOI:** 10.1007/s40820-019-0286-9

**Published:** 2019-07-08

**Authors:** Huan V. Doan, Harina Amer Hamzah, Prasanth Karikkethu Prabhakaran, Chiara Petrillo, Valeska P. Ting

**Affiliations:** 1 Department of Mechanical Engineering, University of Bristol, Bristol, BS8 1TR UK; 2 Department of Oil Refining and Petrochemistry, Hanoi University of Mining and Geology, Duc Thang, Bac Tu Liem, Hanoi, Vietnam

**Keywords:** Metal–organic frameworks, Hierarchical, Macroporous, Composites

## Abstract

Introduction of multiple pore size regimes into metal–organic frameworks (MOFs) to form hierarchical porous structures can lead to improved performance of the material in various applications. In many cases, where interactions with bulky molecules are involved, enlarging the pore size of typically microporous MOF adsorbents or MOF catalysts is crucial for enhancing both mass transfer and molecular accessibility. In this review, we examine the range of synthetic strategies which have been reported thus far to prepare hierarchical MOFs or MOF composites with added macroporosity. These fabrication techniques can be either pre- or post-synthetic and include using hard or soft structural template agents, defect formation, routes involving supercritical CO_2_, and 3D printing. We also discuss potential applications and some of the challenges involved with current techniques, which must be addressed if any of these approaches are to be taken forward for industrial applications.

## Highlights

- The advantages of macroporous metal–organic frameworks (MOFs) in comparison with micro- and mesoporous MOFs are discussed.
- A range of synthetic methods for the fabrication and characterisation of hierarchical MOFs with macroporosity are reviewed.
- The applications, advancements, and challenges of each method are compared and assessed in detail.

## Graphical abstract

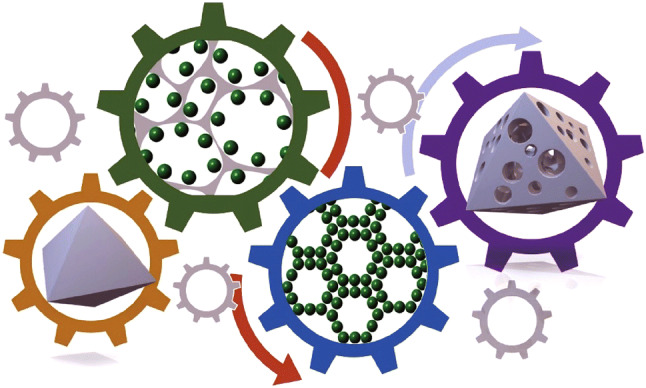

## Introduction

The interactions of gases and liquids with porous materials have been a great source of inspiration for scientists tackling problems related to gas separation [1–4], energy storage [5–8], drug delivery [9–12], and catalysis [13–16]. Pores, which can be discrete or can form complete pathways or channels from one surface to another, play a crucial role in gas adsorption and fluid dynamics [17]. Depending on the size of their pores or channels, materials can be classified as microporous (pore diameters of less than 2 nm), mesoporous (pore diameters between 2 and 50 nm), or macroporous (pore diameters above 50 nm) [18]. There has been a recent trend towards the development of new porous materials incorporating pores with different size regimes to form hierarchical systems with interconnected pores and entirely new properties for desired applications. While the concept of introducing meso- and macropores into microporous materials has been extensively investigated in zeolitic porous systems [19–21], development of metal–organic frameworks (MOFs) with open framework structures comprised of metal nodes and organic ligands has seen a rapid expansion of recent research interest. Advances in synthetic chemistry have reported numerous MOF structures (> 70,000 structures reported so far [22]) with potential use in gas storage [23–25], gas separation [26–29], catalysis [30, 31], carbon dioxide capture [32–34], and as semiconductor materials [35, 36]. As the vast majority of MOF materials are microporous, great interest has developed in the creation of multiple porosities in these materials. A number of excellent reviews on hierarchical MOFs have recently been published [37, 38] and focused on MOFs containing micro- and mesoporosity. The current review emphasises the recent achievements in the development of specifically macroporous hierarchical MOF structures which, though important, are under-represented in the MOF field. Macropores are desirable in hierarchical porous materials for enabling faster molecular diffusion and mass transfer, which is especially important for applications involving large molecules, viscous solutions or applications involving high throughput with low pressure gradients.

The benefits of a hierarchical structure for catalytic applications have been well established in purely inorganic systems. For example, hierarchical mesoporous–macroporous structures in high internal phase emulsion (HIPE) template halloysite nanotubes showed greatly improved catalytic activity for the conversion of cellulose to 5-hydroxymethylfurfural because of the catalyst’s increased permeability and mass transfer efficiency [39]. Similarly, the macroporous nature of the support in hierarchical flower-like TiO_2_ superstructures helps in facilitating more efficient photo-degradation of large dye molecules such as methyl orange [40]. The degradation of Rhodamine B under visible light irradiation using hierarchical core–shell Fe_3_O_4_/WO_3_ was tested by Xi et al. [41], showing that the combination of mesoporous and macroporous regions enhances the photocatalytic activity with almost 100% decomposition achieved after 90 min compared to < 20% and 50% for the iron(iii) oxide core and tungsten(vi) oxide, respectively. In this case, the large voids between the Fe_3_O_4_ and WO_3_ in the hierarchical structure were deemed to synergistically reduce the recombination rate of photogenerated electron–hole pairs, further improving the photocatalytic degradation. The importance of pore size in directing catalysis was further illustrated by Feng et al. [42] using a hierarchical MOF composite comprised of PCN-222 (also known as MOF-545) and ZIF-8 to achieve size-selective catalysis. The PCN-222(Fe)@ZIF-8 composite was exposed to two molecules sensitive to oxidation by PCN-222, namely *o*-phenylenediamine (*o*-PDA) (0.5 × 0.5 nm) and 2,2′- azino-bis(3-ethylbenzothiazoline-6-sulphonic acid) (ABTS) (0.7 × 1.6 nm). While PCN-222 successfully catalysed the transformation of *o*-PDA, the catalysis of the larger ABTS molecule was hindered due to constricted diffusion through the small pore size window of ZIF-8. Thus, considering the added value of hierarchical structure in these inorganic systems, the ability to fabricate hierarchically porous MOF structures may be similarly beneficial when considering future practical applications.

The nature of the porosity in a material can be determined by various characterisation techniques, which can be generally grouped into use of fluid penetration, scattering, and imaging. Fluid penetration techniques involve the filling of pores with a liquid or a gas, to obtain information on the pore sizes and volumes. For MOFs (conventionally microporous materials), gas sorption (generally with nitrogen at 77 K up to 1 bar pressure) is the most common characterisation method to establish the specific surface area, pore volume, and pore size distributions. To obtain this information, the gas sorption isotherms are modelled using standard and widely accepted approaches such as the Brunauer–Emmett–Teller (BET) method for calculating surface area [43], density functional theory (DFT) and the Horvath–Kawazoe (H–K) method for calculation of the pore size distribution in the micropore region, and the Barrett–Joyner–Halenda (BJH) for mesopore distribution [44, 45]. However, nitrogen sorption at 77 K will typically not provide information on macropores larger than 100 nm [44]. For materials containing pores in the mesopore and macropore range (~ 4 nm–60 µm), an alternative method is mercury intrusion porosimetry, which uses non-wetting mercury to penetrate the pores under pressure [44, 46]. While this method can produce a macroporous size distribution in robust porous materials, it has the disadvantages of using a toxic compound (mercury) for the characterisation, cannot be used on soft or deformable structures, and (unlike gas sorption) is destructive to the sample.

Scattering techniques, which involve bombarding the sample with, for example, X-rays, neutrons or electrons to obtain a pattern, can be non-destructive while still enabling characterisation of the porosity. While powder X-ray diffraction (PXRD) and single-crystal X-ray diffraction (SXD) are commonly used to study the microstructures of crystalline porous materials, small angle X-ray scattering (SAXS) can be used to probe the variations in scattering length density which occur over distances exceeding typical interatomic spacing to determine larger particle or pore sizes. Hence, SAXS can be used to calculate pore size distributions in porous materials (1–100 nm range) and can be used for both crystalline and non-crystalline materials, and for materials having regular (but perhaps non-crystalline or disordered) porosity [47]. Both neutron scattering and X-ray scattering can be used to provide information on the pore dimensions in a bulk sample, unlike fluid penetration, which can only give information on interconnected pores that are accessible to penetration by the fluid (“open” porosity), scattering can provide information on isolated or “closed” pores in the material. However, for scattering techniques to be used for characterisation of porosity, there needs to be appropriate scattering length contrast between the pores and the surrounding material, which can be difficult in the case of X-ray scattering from MOFs, where the voids are often filled with air and the materials contain predominantly light organic materials such as C or H. Increased contrast may be achieved by the filling of the pores with a suitable contrast agent [48], which may have the undesirable effect of distorting the pores, for example, in the case of flexible MOFs, or where there is a soft, compliant matrix, such as a hydrogel. In specific cases, where the scattering length density contrast is too small for X-rays, small angle neutron scattering (SANS) can be used [49]. As neutron scattering length varies independent of atomic number, even light atoms such as hydrogen and carbon can be easily distinguished using neutrons [50], but the need for a neutron source means this is a far less widely used technique.

As can be seen throughout the literature, imaging is by far the most common technique to visualise the macropores appearing in MOFs due to widespread accessibility of the technique and ease of use. Microscopy can provide valuable insight into the shape and spatial distribution of pores. For small macropores (e.g. below 10 µm), scanning electron microscopy (SEM) and transmission electron microscopy (TEM) can be used to image porosity and can be performed in a very straightforward way on a very small amount of sample. However, these imaging methods are not bulk techniques (with SEM in particular being restricted to definition of surface features) and can only observe a small number of particles and therefore may not be representative of the bulk porosity. For imaging of larger macropores (from a few tens of microns to millimetres in size), optical microscopy can be useful, but cannot provide information on the connectivity or tortuosity of the interior pore network in monolithic structures. The ability of X-rays to penetrate light materials can be used in X-ray computed tomography (CT), whereby a series of 2D X-ray images of a material are computationally compiled and reassembled to obtain a 3D image [51]. X-ray CT can thus be used to image large pores in monolithic structures, though the resolution of such techniques is currently limited to a few tens of microns [51]. Thus, it can be seen that few characterisation techniques can span the full range of pore sizes. Therefore, as will be shown in this review, due to the complexity of such materials, a combination of several techniques may be needed to obtain a full description of the macroporosity in hierarchical MOF structures.

The routes to formation of such complex structures are equally varied. In terms of approaches to increase pore dimensions in MOFs using synthetic chemistry, the typical methods used in MOF synthesis to produce mesopores (such as extending ligand lengths or enlarging building blocks) are of limited use in the creation of much larger macropores. This is due to the difficulties in stabilising these pores against collapse upon desolvation during activation [52]. Hence, the formation of macroporosity (rather than mesoporosity) in MOFs necessitates the use of very different fabrication approaches. In this review, we focus on the synthetic strategies and challenges of creating MOFs with pore structures containing macropores and provide readers with a wider scope of the strategies available for fabrication of hierarchical macroporous MOFs.

**Fig. 1:**
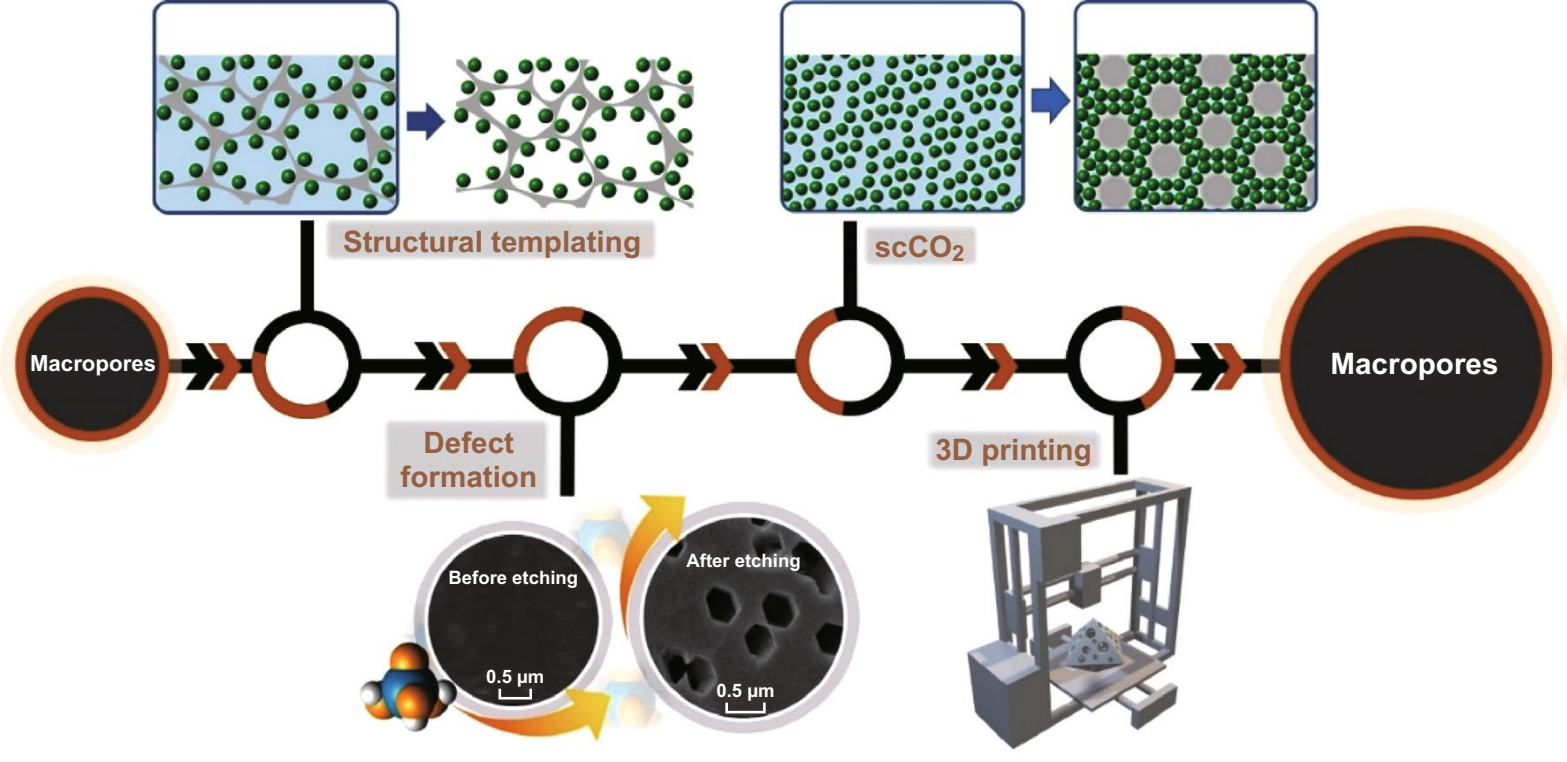
Schematic representation of synthetic methods for hierarchical MOFs with macroporosity

We grouped the macroporous MOF structures that have been studied under four broad approaches, which are presented in order of most commonly reported method to least reported as follows: macroporous MOFs synthesised via structural templating, defect formation, use of compressed or supercritical CO_2_ (scCO_2_), and 3D printing routes (as summarised in Fig. 1 and Table 1).

**Table 1:** A summary of preparation techniques, crystallisation routes, and templates for the formation of hierarchical MOFs with macroporosity

Preparation techniques	Macropore formation route	Templates, substrates, modulators or reagents used	MOFs prepared using these methods	References
Hard structural templating	Direct synthesis	Macroporous carbon derived from kenaf stem, polymer-derived ceramic (PDC) foam, oxide-bonded silicon carbide and alumina (Al_2_O_3_), ceramic foams (CF), copper foam	HKUST-1, CAU-10, UiO-66 (Zr), MOF-5	[59, 62, 63, 66]
	Post-synthetic treatment	Nickel foam	MIL-101 (Cr)	[60]
Soft structural templating	Direct synthesis	Cellulose solution, konjac glucomannan, graphene oxide, melamine foam (MF), three-dimensional polystyrene (PS), N,N-dimethyloctylamine (DMOA), polyacrylamide (PAAm)	ZIF-8, ZIF-9, ZIF-12, PCN-224, HKUST-1	[69, 70, 74–78]
	Post-synthetic treatment	Melamine sponge	ZIF-67	[71]
Defect formation	Direct synthesis (linker modulation)	Alkyl chains, monocarboxylic acids	MOF-5, UiO-66	[92, 93]
	Post-synthetic treatment (acid etching)	Phenolic acid, cyanuric chloride and tetraethylamine (TEA), phosphoric acid, hydroquinone, H_3_BO_3_ and NaCl	ZIF-8, IRMOF-3, MIL-101(Fe), HKUST-1	[94–96, 98, 99]
Use of compressed or supercritical CO_2_	Post-synthetic treatment (scCO_2_ drying)	Emulsion	HKUST-1, AlBTC, AlBDC	[109, 110]
	Direct synthesis (expanded solvent)	N-EtFOSA/TMGT solution, DMF, DMSO/MeOH	Zn-BTC, HKUST-1, Co-BTC	[113–115, 120]
3D printing	Post-synthetic incorporation	Polyvinyl alcohol (PVA), trimethylolpropane propoxylate triacrylate (TMPPTA), polylactic acid (PLA)	MOF-74 (Ni), UTSA-16 (Co), UiO-66, ZIF-8	[146–148]
	Direct synthesis	Anionic 2,2,6,6-tetramethylpiperidine-1-oxylradical-mediated oxidised cellulose nanofibers (TOCNFs)	ZIF-8 and MIL-100 (Fe)	[149]

Structural templating involves use of a template containing macroporous voids to produce a macroporous composite structure by directly growing the crystalline MOF either on or around a template. The template can be “hard” (e.g. a foam or porous monolith) or “soft” (e.g. a gel or emulsion) and can be retained or removed after templating.

Defect formation describes the disruption of the periodic crystalline structure of the MOF e.g. by incorporating a sizeable ligand during synthesis which introduces a porous defect or by selective removal of sites to produce pores, e.g. via post-synthetic acid etching of MOF single crystals.

Compressed or supercritical CO_2_ has been used extensively for activation of MOFs [53] to remove solvents and soft templates to produce porous aerogel structures, but scCO_2_ has also recently been shown to be useful in expanded solvent systems for producing macroporous voids during direct synthesis of MOFs.

The recent use of 3D printing combines MOFs with binders to create MOF-based inks capable of forming hierarchical monoliths on deposition. This represents a new method with potential to enable the construction of hierarchical monoliths with complex geometries.

Most of these methods can be employed either using MOF precursor solutions (termed “direct synthesis”) or using pre-formed MOFs (“post-synthetic treatment”), as shown in Table 1.

## Preparation Techniques for Hierarchical MOFs with Macroporosity

### Structural Templating Method

The most common approach to the introduction of macropores into an intrinsically microporous MOF is using structural template to form a hierarchical structure. As demonstrated for a multitude of other porous material systems, the macroscopic shape and the pore size of the resulting hierarchical structures can be well controlled through judicious choice of template [54–58]. The resulting hierarchical porous MOF structures could potentially exhibit improved properties such as more rapid molecular diffusion compared to the respective non-hierarchical MOFs, better mechanical or thermochemical stability, and immobilisation of nanoparticulates. Thus, they are preferentially used in some applications that require easy integration and regeneration (e.g. for adsorbent systems [59, 60]) or exceptional mass transfer (e.g. for catalyst supports [61]).

The macroporous MOF structures deriving from the template approach can be divided into two main categories: one is that the template remains in the final MOF structure, forming a composite material, and the other is that the template is sacrificial and removed to obtain a macroporous structure in the pure MOF (often called a MOF aerogel or a foam). Templates can be broadly grouped into “hard” and “soft” structural templates.

#### Hard Structural Templating

Hard templates may be carbons [62], ceramics [59, 61, 63–65], and metals [60, 66–68]). They are typically in the form of monoliths, porous membranes, or foams [60, 62, 69]. Because hard templates are difficult to be removed, the majority of these templates act as supports, resulting in macroporous composite MOF structures.

**Fig. 2:**
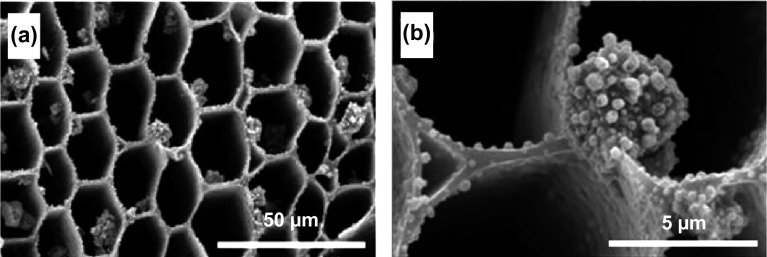
SEM images of HKUST-1/3D-KSCs_800_ at different magnifications, showing the growth of microporous HKUST-1 crystals on the 3D-KSCs_800_ macroporous template. Reprinted with permission from Ref. [62]. Copyright 2018 Elsevier B.V

As an example of direct MOF synthesis on a hard template to obtain a hierarchical structure with macropores, Xie et al. [62] grew HKUST-1 MOF crystals on three-dimensional kenaf stem-derived macroporous carbons (3D-KSCs), forming a composite material with a macroporous structure. In this work, it was shown that a large number of smooth-faced octahedral HKUST-1 crystallites with maintained morphology were formed on the surface and inner walls of the macroporous 3D-KSC template (Fig. 2a, b), with the amount of HKUST-1 loaded onto the template increasing with increased reaction time. The as-obtained composites were tested for electrochemical sensing of glucose, showing very promising activity with a relatively wide linear range (15.84 µM–5.62 mM), low limit of detection (4.8 µM), and a high sensitivity (28.67 µA mM^−1^ cm^−2^). The excellent electrocatalytic activity was attributed to the presence of macropores in the hierarchical structure which was led to improved mass transfer of the glucose. Using a similar hard template, Sandra et al. [59] synthesised a series of silicoboron carbonitride (Si/B/C/N) polymer-derived ceramic (PDC) foams with micro-/macroporosity using silicon and carbon high internal phase emulsions (HIPE) as exo templates and boron-modified polycarbosilazane as an organosilicon precursor. HKUST-1 was impregnated by growing the MOF inside the macropores of the PDC to form Si/B/C/N@MOF composite foams, allowing faster and more efficient uptake and release kinetics, in particular for CO_2_ capture.

**Fig. 3:**
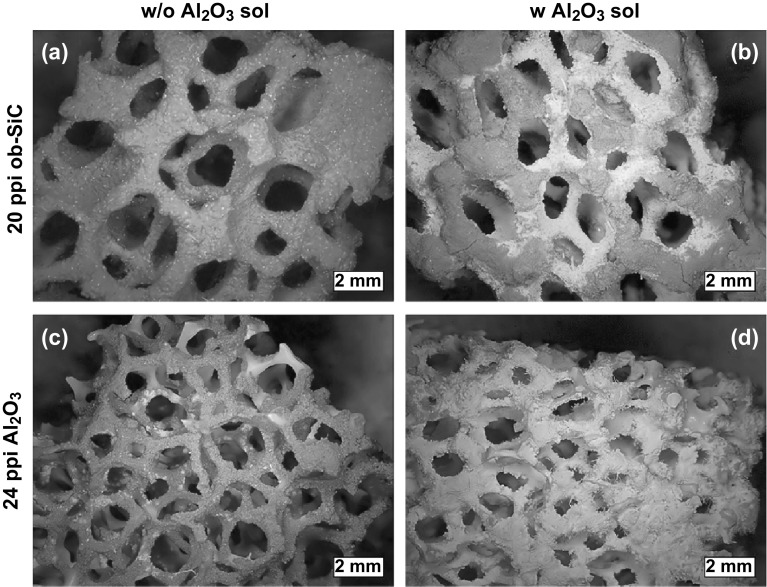
SEM images of HKUST-1 coatings on ob-SiC and Al_2_O_3_ ceramic foams **a, c** without and **b, d** with preliminary alumina sol coating. The HKUST-1 coating layers on the ob-SiC composites are thicker than those on Al_2_O_3_ composites. Reprinted with permission from Ref. [63]. Copyright 2016 Elsevier Inc

Direct synthesis of MOFs on hard templates has also been shown by Betke et al. [63] and to be readily adaptable to a variety of MOF types, with a range of macroporous MOF@ceramic foam composite materials being formed by growing MOFs such as HKUST-1, CAU-10, MIL-101(Cr), and UiO-66(Zr) on macroporous oxide-bonded silicon carbide (ob-SiC) and alumina ceramic foams (CF). In these cases, the MOFs were being tested for use in sorptive heat pump/heat storage applications; the macroporous ceramic structure not only had benefits for the mass transfer and adsorption/desorption kinetics (which had the effect of decreasing cycling times), but also enhanced heat transfer in comparison with the powdered MOF, increasing the potential for adsorptive cooling. The surface properties of the CF were modified by silanisation using aminopropyl triethoxysilane/terephthaloyl chloride treatment prior to the MOF crystallisation to obtain a thick homogeneous MOF coating on the surface. The thickness of the MOF on the surface was also influenced by an alumina sol coating on the cellular CF support before silanisation, as supported by the SEM images of HKUST-1 on the surface without (Fig. 3a, c) and with (Fig. 3b, d) alumina sol coating. The coating thickness of HKUST-1 crystals ranged between 112 and 264 µm for the ob-SiC-based composites and between 36 and 151 µm for the HKUST-1@alumina composites. Similarly, Hu et al. [66] reported a facile synthesis of hierarchical porous MOF-5 structure using a copper foam as a template, for removal of volatile sulphides from plant sources. The MOF composite was prepared by solution impregnation of zinc nitrate and terephthalic acid into a pre-treated copper foam. The macroporous MOF monolith was tested for the extraction of volatile organic sulphur compounds with detection values of 6.0–54.6 µg g^−1^.The extraction process could be replicated at least 200 times, indicating that the formation of the macroporous composite structure may have been beneficial for the stability of MOF-5, which is widely recognised for being prone to hydrolysis, particularly in humid environments.

**Fig. 4:**
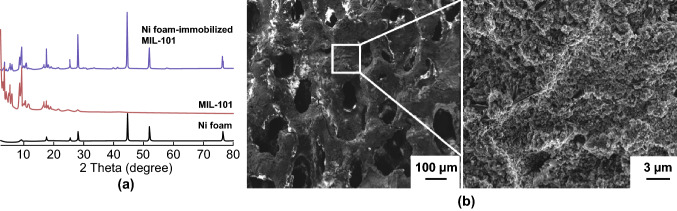
**a** PXRD patterns of Ni foam-immobilised MIL-101(Cr) (blue) showing the presence of peaks which correspond to both Ni foam (black) and MIL-101(Cr) nanocrystals (red). **b** SEM images of Ni foam-immobilised MIL-101(Cr) showing the presence of multi-layered MIL-101(Cr) nanocrystals on the macroporous Ni foam. Reprinted with permission from Ref. [60]. Copyright 2015 Elsevier B.V. (Color figure online)

While the direct crystallisation of MOFs onto a macroporous template is the most commonly used method, in addition to the direct synthesis and growth approaches discussed above, a microporous MOF can also be endowed with a macroporous structure by combining a macroporous substrate with a pre-formed MOF. For example, Ren et al. [60] prepared MIL-101(Cr) powders and immobilised the nanocrystals on a macroporous nickel (Ni) foam via spray coating. The PXRD pattern of the MIL-101/Ni foam composite showed peaks that corresponded to both MIL-101 powders and Ni foam (Fig. 4a). The incorporation of the MOF crystals on the Ni foam was further evidenced by SEM images, in which the porosity of the Ni foam was reduced by the presence of multi-layered MIL-101 powders on its surface (Fig. 4b). The MIL-101/Ni foam composite was tested for hydrogen storage and the composite with 81 wt% loading of MIL-101(Cr) nanocrystals exhibited a hydrogen adsorption capacity similar to pure MIL-101(Cr) powders. Although an increase in hydrogen uptake was not observed, the immobilisation of MIL-101(Cr) powders onto a macrostructural support provides a facile method to process MOF powders and immobilises the nanoparticulates for applications in practical systems.

#### Soft Structural Templating

**Fig. 5:**
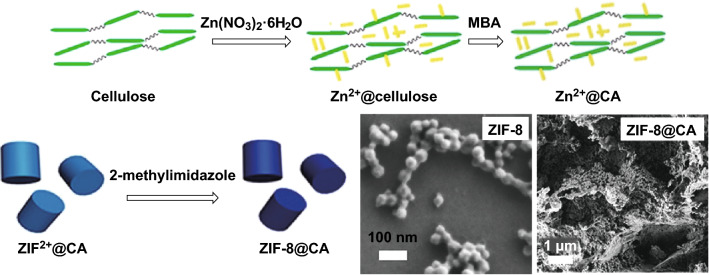
Schematic illustrations showing the preparation of ZIF-8@CA and the resulting SEM images. Reprinted with permission from Ref. [74]. Copyright 2018 Elsevier Inc

**Fig. 6:**
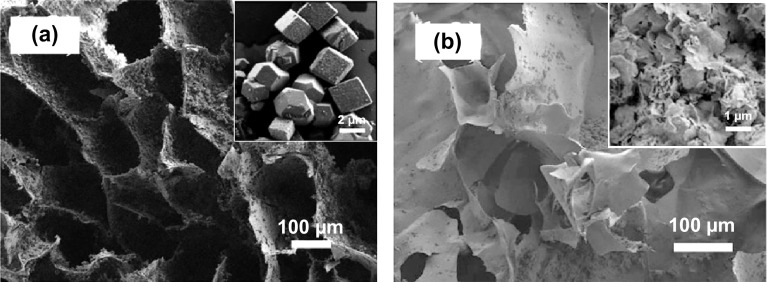
SEM images of **a** ZIF-9@CA and pure ZIF-9 inset, and **b** ZIF-12@CA and pure ZIF-12 inset. Reprinted with permission from Ref. [75]. Copyright 2018 Elsevier B.V

In situations where flexibility of the porous support is desired, soft macroporous templates are sometimes advantageous. Soft templates include surfactants [70], polymers [71–73], gels, and emulsions. Flexible macroporous composite materials were developed by Bo et al. [74]; they reported a facile method for the direct synthesis of a cellulose hybrid aerogel of ZIF-8 (ZIF-8@CA), in which zinc cations were dispersed in a cellulose solution before prompting nucleation reaction with 2-methylimidazole to form the MOF as illustrated by the schematic in Fig. 5. ZIF-type materials are favoured candidates because of their high water stability which is crucial for gel formation without losing the original crystallinity. As shown in the inset of Fig. 5, the resulting hybrid material retained the original ZIF-8 particle size (20–40 nm) with hierarchical porous structures (pore size between 10 and 100 µm). Cr(VI) sorption was performed on this sample, showing a greatly improved uptake in the composite material when compared with a single component. A similar synthetic method was applied to the synthesis of ZIF-9 and ZIF-12 on CA (Fig. 6a and b, respectively) [75], which showed macropores with improved catalytic performance in p-nitrophenol degradation (90% in 1 h). Preparation of a ZIF aerogel via a one-pot strategy was further explored using various templates such as biodegradable konjac glucomannan by deacetylation [76] and graphene oxide [77] and resulted in structures with outstanding photoelectric, mechanical, and thermal properties.

**Fig. 7:**
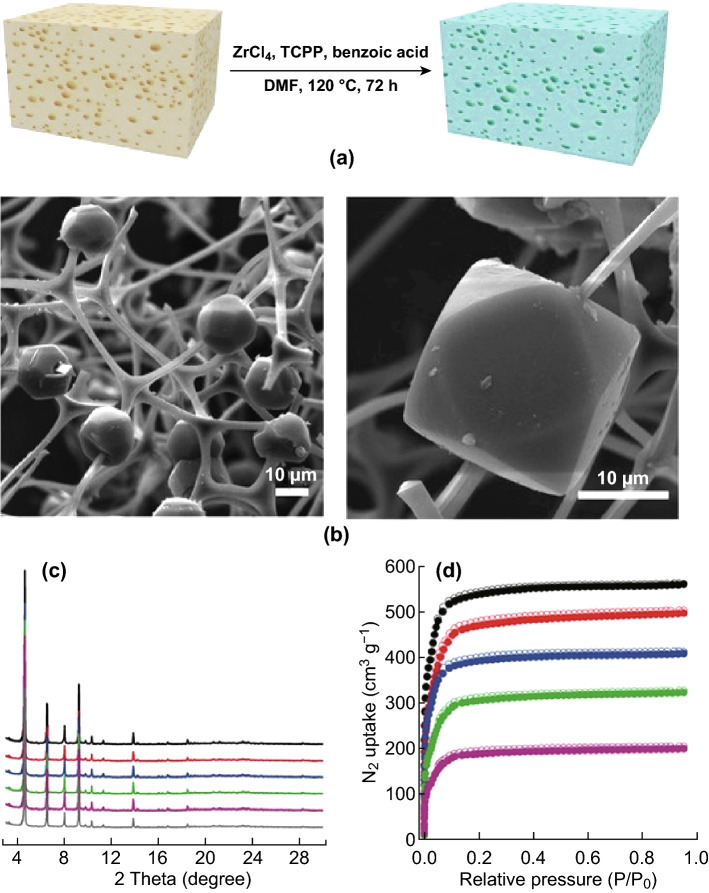
**a** Procedure for the preparation of PCN-224 decorated melamine foam composites. **b** FESEM images of PCN-224(Fe)/MF composite depicting the melamine foam network homogeneously decorated with PCN-224(Fe) microcrystals. **c** The high similarities between the PXRD patterns of PCN-224(Fe) (grey), PCN-224(Fe)_50%_/MF (purple), PCN-224(Fe)_100%_/MF (green), PCN-224(Fe)_150%_/MF (blue), PCN-224(Fe)_200%_/MF (red), and PCN-224(Fe)_325%_/MF (black) indicating the structural stability of the MOF composites. **d** N_2_ sorption isotherms of PCN-224(Fe)_50%_/MF (purple), PCN-224(Fe)_100%_/MF (green), PCN-224(Fe)_150%_/MF (blue), PCN-224(Fe)_200%_/MF (red), and PCN-224(Fe)_325%_/MF (black). The BET surface areas increased with the increasing loading amount of PCN-224(Fe) in the composites. Reprinted with permission from Ref. [78]. Copyright 2018 Wiley-VCH Verlag GmbH & Co. KGaA, Weinheim. (Color figure online)

Another example of using soft structural templates in direct synthesis was given by Huang et al. [78], who reported a series of porous coordination network (PCN) MOFs that were integrated into a macroporous monolithic melamine foam (MF) using a one-pot synthesis (Fig. 7a, b), resulting in hierarchical porosity, and a flexible and elastic texture, which was favourable for heterogeneous catalysis. Additionally, the BET surface areas were found to increase with the loading amount of PCN-224(Fe) in the composites (1623–1704 m^2^ g^−1^) (Fig. 7d). The positive effect of the macroporous structure on the application of these samples was confirmed in the highly efficient catalytic epoxidation of unsaturated cholesteryl esters. The 2 mol% of PCN-224(Ru) 200%/MF showed an excellent yield (92%) of the corresponding epoxide after 36 h, compared to a yield of 56% using normal PCN-224(Ru) and 0% conversion using a control of pure melamine foam.

**Fig. 8:**
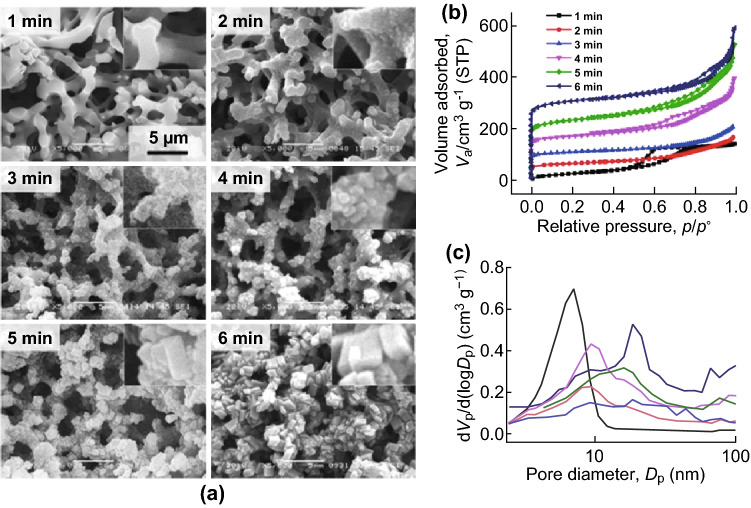
**a** SEM images of the conversion of Cu-06 to HKUST-1 with 1-min time intervals, showing the growth of polyhedral crystallites with the preservation of the co-continuous macroporous gel structure. All the images are in the identical magnification. **b** N_2_ sorption isotherms and **c** BJH pore size distributions with respect to increased immersion times. The BET surface areas and pore sizes increased with immersion times. Reprinted with permission from Ref. [79]. Copyright 2015 Royal Society of Chemistry

Moitra et al. [79] developed an innovative protocol for the direct synthesis of a macroporous monolithic HKUST-1 composite by conversion of Cu(OH)_2_-based networks in a polyacrylamide (PAAm) gel. The presence of PAAm in the original (oxy) hydroxide network supported the scaffold of Cu(OH)_2_ by chelating the copper ions with the N-groups in the amide, resulting in gelation and producing a co-continuous network which was converted to HKUST-1 through coordination by H_3_BTC. The macropore size within the aggregated particles could be controlled by changing the amount of PAAm from 0.3 to 1 g or by varying the immersion time in H_3_BTC between 3 and 6 min (Fig. 8a). In addition, enhancements in N_2_ uptake (Fig. 8b) and pore size (Fig. 8c) were reported with increased immersion times, corresponding to higher MOF loadings. The resulting HKUST-1 monolith showed high crystallinity, a high surface area of 1315 m^2^ g^−1^, and good mechanical properties (stress values at catastrophic failure of 1.5 MPa), which are promising for possible applications in continuous flow reactors, which require both high mass transfer and immobilisation of the MOF.

**Fig. 9:**
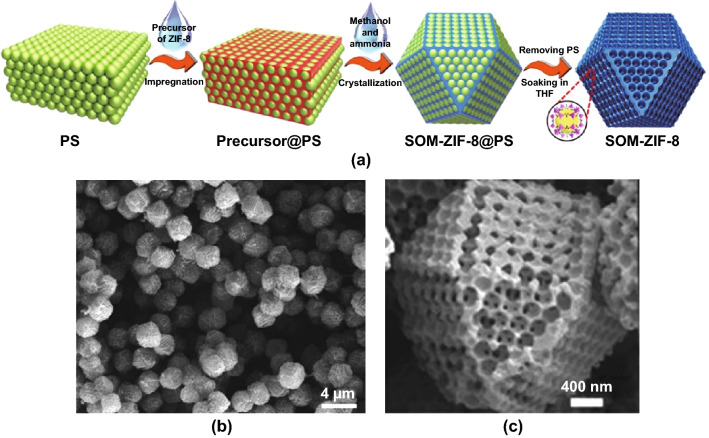
**a** Schematic diagram of SOM-ZIF-8 synthesis. SEM images of **b** SOM-ZIF-8 and **c** an isolated crystal of SOM-ZIF-8 showing the tetrakaidecahedron morphology. Reprinted with permission from Ref. [69]. Copyright 2018 The Authors, some rights reserved

Soft templates are also beneficial where facile template removal is desired. As an example, a polymer has been used as a sacrificial soft template to form a macroporous MOF, and a hierarchical SOM-ZIF-8 MOF was formed by impregnating ZIF-8 precursors into the interstices of three-dimensional polystyrene (PS) spheres in a mixture of methanol/ammonia (Fig. 9a) [69]. SEM image confirmed that ZIF-8 single crystals were uniformly grown around the PS template and the overall network displayed a tetrakaidecahedron morphology (Fig. 9b, c). A highly structured macroporous framework could be accessed by dissolving the PS template in THF and subsequently activated via removal. The presence of macropores in the MOF was confirmed by mercury intrusion porosimetry, and the size of the macropores could be modified (80–470 nm) by changing the diameter of the polystyrene templates. Furthermore, Shen et al. reported that the single-crystal growth was governed by the solvent system and the crystal size was conveniently tuned by modifying the ratio of the methanol/ammonia mixture.

Duan et al. [70] reported the soft-templated synthesis of a HKUST-1 framework that contained micro-, meso-, and macropores, using a *N*,*N*-dimethyloctylamine (DMOA) surfactant as a sacrificial template. The role of the DMOA as a template was demonstrated by mesodynamics simulation in which the introduction of DMOA into the reaction mixture resulted in the formation of supramolecular micelles. Subsequently, the MOF precursors [i.e. a mixture of hydroxy double salt (Zn and Cu)] and benzenetricarboxylic acid (H_3_BTC) were added and self-assembled on the surface of the template micelles at room temperature and then formed the hierarchical porous HKUST–1 upon the removal of DMOA template. SEM images revealed the hierarchical nature of this MOF whereby continuous pore voids were formed between the nanoparticles. TEM further confirmed the abundance of mesopores and macropores (pore sizes of 40–100 nm) in the final product. Because of the presence of macropores which contribute to high molecular diffusion, the hierarchical MOF exhibited a high toluene storage capacity (646 mg g^−1^ at 298 K), representing a 25 wt% increase compared to that for the non-hierarchical HKUST-1 (516 mg g^−1^) [80]. This value is also much higher than other microporous MOFs and zeolites [81–83]. The use of surfactants in the preparation of such hierarchical MOFs introduces a new way to allow the degree of porosity to be readily tuned by adjusting the amount and type of surfactant used.

**Fig. 10:**
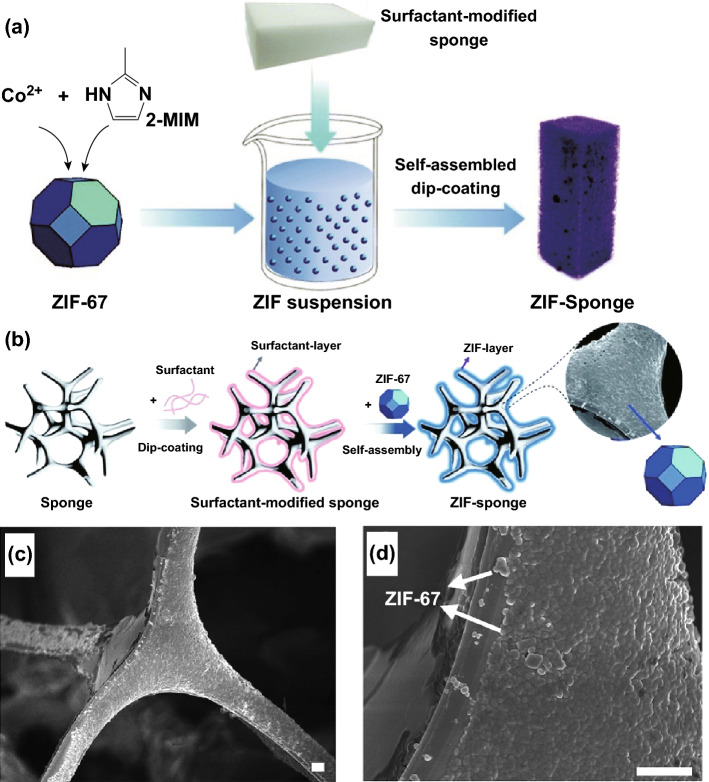
Schematic illustrations showing the preparation of ZIF–sponge, showing **a** surfactant-assisted dip-coating self-assembling process and **b** surface modification of the sponge skeleton first with a surfactant and then ZIF-67. **c, d** SEM images of ZIF–sponge prepared with the assistance of SDBS under different magnifications (the scale bar is 2 µm), showing ZIF nanocrystals were dip-coated on the surface of melamine sponge. Reprinted with permission from Ref. [71]. Copyright 2015 Royal Society of Chemistry

In addition to such direct synthesis methods, it may also be the case that pre-formed MOFs can be deposited onto a macroporous soft template using post-synthetic treatment. For example, Lin et al. [71] used a surfactant-assisted dip-coating method to synthesise a flexible three-dimensional hierarchically porous ZIF-67/melamine sponge composite (as illustrated in Fig. 10a, b). The ZIF nanocrystals were dip-coated on the surface of a surfactant-modified pliable melamine sponge by immersing it in ZIF-67 suspension while stirring for 3 h. The self-assembly of MOF crystals on the modified melamine sponge occurs via electrostatic attractions and possibly *π*–*π* stacking interactions. The SEM image (Fig. 10c, d) showed that the clean, smooth surface of the melamine sponge became roughened by the ZIF-67 coating. Four types of surfactants were used for the modification of the melamine sponge including anionic sodium dodecyl benzene sulphonate (SDBS), cationic cetyltrimethylammonium bromide (CTAB), non-ionic hydrophobic sorbitan monooleate (denoted as SPAN-80), and non-ionic hydrophilic Triton X-100 to study the effect of surfactant properties on the ZIF-67 loading. It was found that increasing the concentration of CTAB and Triton X-100 did not increase the ZIF-67 loading. However, an increase in ZIF-67 loading was observed when the melamine sponge was modified by SDBS and SPAN-80. According to zeta potential measurements, in the testing pH range, Triton X-100 was found to be neutral, ZIF-67 and CTAB displayed positive surface charges, and SDBS and SPAN showed negative surface charges. The increase in ZIF-67 loading with SDBS and SPAN surfactants was attributed to the electrostatic attraction between the positively surface charged ZIF-67 and negatively surface charged SBDS and SPAN. This approach of synthesising the ZIF-67/melamine sponges could be further used to fabricate various shapes such as fibres or membranes and can be used as adsorbents for the removal of toxic large molecule pollutants like malachite green dye.

The use of structural templating agents remains the most established method in the formation of macroporous MOFs due to the wide range of templates and templating agents that can be employed in this technique. As can be seen by the examples given thus far, the resulting hierarchical structures generally adopt the shape of the templating agents. It was also shown that these templates could be introduced either in the reaction solution during MOF synthesis or in the precursor solution where the prepared MOFs were dispersed. The final materials could be obtained as MOF-based composites with the template retained or as pure MOF superstructures without the template. When the templating agents are incorporated to produce the final composite MOF structures, in addition to the immobilisation of the MOF crystals, they may also contribute to improved mechanical and thermochemical stability of the MOF composites. In the case of MOFs with sacrificial templates, washing processes are required to remove the templates. In some cases (such as coordination polymer frameworks, silicates and MCM-41), it can be detrimental to the stability of the pore structures [84–87]. Furthermore, incomplete removal can lead to occlusion of the pores and therefore loss of functionality for gas sorption/catalytic reactions. In terms of potential uses, the MOFs formed via this method have been utilised in various applications from catalytic applications, heat and mass transfer to extraction of volatile compounds.

### Defect Formation Method

From the point of sustainable processing, the ability to form macroporosity in the absence of sacrificial templates or scaffolds would be preferable. A commonly used strategy for obtaining hierarchical porous MOFs without using templates is through direct introduction of defects into the microporous structures. This process could be carried out using either in situ synthetic methods (e.g. using linker modulation) or using post-synthetic treatment (e.g. via acid etching). Linker modulation and linked labilisation methods predominantly produce mesoporous defects in microporous MOFs. Yuan et al. demonstrated using a pro-labile organic linker for the synthesis of PCN-160 MOF. The defect in the pre-formed MOF was introduced by splitting this pro-labile linker into removable monocarboxylate moieties under acidic conditions, which improved their gas adsorption and catalytic properties [88]. Similarly, Kim et al. created mesoporous defects in microporous HKUST-1 using an acetic acid fragmented linker co-assembly method. A small amount of acetic acid removed the BTC linker to create mesoporous defects. On increasing the amount of acetic acid, metal clusters in the MOF were removed, which resulted in interconnected defect sites and mesoporous channels inside the MOF, which enhanced the methane storage capacity in the defective MOF [89]. These methods have been employed extensively in numerous studies to functionalise meso- and macropores with straightforward synthetic procedures and a number of effective mechanisms have been demonstrated [90, 91], but there are now additional reports of using similar methods for the production of macroporous structures.

**Scheme 1:**
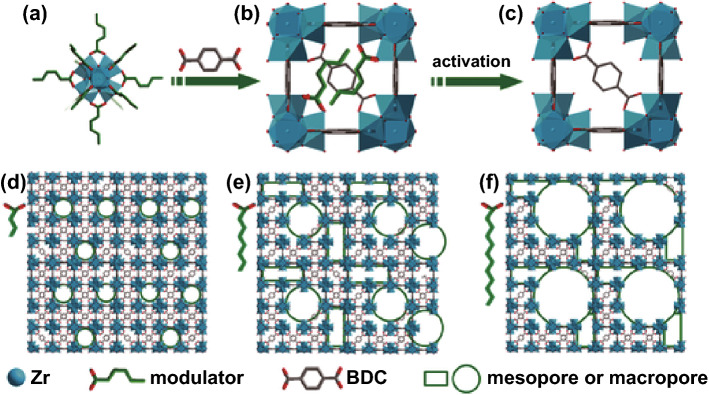
Schematic illustration of the synthesis of HP-MOFs with adjustable porosity using UiO-66 as an example. Reprinted with permission from Ref. [93]. Copyright 2017 Wiley-VCH Verlag GmbH & Co. KGaA, Weinheim

Examples of macroporous materials formed by in situ linker modulation methods include the introduction of extended aliphatic chains to MOF linkers to obtain sponge-like or pomegranate-like crystallite structures in MOF-5 [92] and using alkyl chains as modulators for macropore formation in UiO-66 [93]. In the case of the hierarchical macroporous MOF-5 structures, the formation of either the sponge-like (spng-MOF-5) or pomegranate-like (png-MOF-5) structures was the result of incorporating different proportions of the linkers containing the large void-forming alkyl chain (50% and 30% incorporation, respectively). The modulators were shown by gas sorption to prompt formation of additional mesoporosity and macroporosity into MOF-5, which influenced the CO_2_ uptake properties of the different structures. However, the additional beneficial effects of having such an interconnected macroporous structure which was evidenced from the SEM would have been more obvious if a bulkier adsorbent than CO_2_ had been employed. In the case of the defective UiO-66 synthesised by a modulator-induced defect formation strategy described by Cai et al. [93], Zr^4+^ ions reacted with less than stoichiometric amounts of BDC^2−^ linkers and COO^−^ ions in monocarboxylic acids as a modulator to pre-coordinate Zr-oxo clusters. The alkyl chain on the modulators linked these clusters to create large voids during synthesis (Scheme 1). The pore sizes were tuneable by changing alkyl chain length and the molar ratio of Zr to H_2_BDC. The ability to transport large molecules through the defect structure was demonstrated using the catalytic methanolysis of styrene oxide. The results indicated that the hierarchical porous UiO-66 with additional mesopores exhibited superior catalytic activity when compared to the normal MOF. It was foreseen by the authors that macropores could be obtained with further expansion of the alkyl chain length in the modulator. However, they noted that the high *pK*_*a*_ values of elongated alkyl chains could decrease the solubility and coordination ability of the modulator with Zr-oxo clusters, which might result in adverse effects such as pore shrinkage.

An alternative approach to direct synthesis of defective MOFs with macropores is via the post-synthetic etching of prepared microporous MOFs. This can be done either by an acid etching mechanism whereby selected acid molecules diffuse through the MOF structure, forming large voids over time, or by synergistic etching where bulky acid molecules modify and protect the MOF surface to obtain the holey materials.

Koo et al. [94] investigated a size-selective acid diffusion method for the post-synthetic treatment of MIL-100 (Fe) using phosphoric acid (*d* = 0.61 nm) as the etching agent. During the etching process, phosphoric acid could specifically diffuse through the hexagonal windows (*d* = 0.89 nm) but not through the pentagonal windows (*d* = 0.49 nm) of MIL-101(Fe) to form macropores in the framework, preserving the microporous nature by selective removal of metal nodes and BTC linkers. The framework crystallinity and morphology of the MOF crystals were well maintained even after post-synthetic treatment. Note that this process cannot be achieved by employing smaller acids such as HCl (*d* = 0.34 nm) in the same manner. This is simply because the small cages could also be attacked during etching, leading to complete collapse of the MOF structure. This etching method seems to be effective for the creation of hierarchical porous MOFs with tuneable pore sizes. However, as mentioned in the literature [94], these approaches are only applicable for highly water-stable MOFs.

**Fig. 11:**
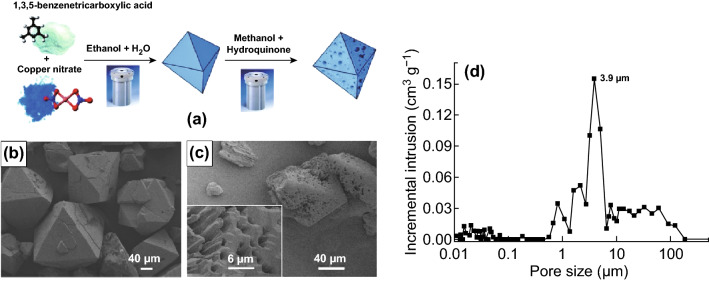
**a** Schematic representation for the preparation of HKUST-1 particles and the subsequent modification to produce macroporous HKUST-1 particles. **b** SEM image of HKUST-1 microparticles prepared by solvothermal synthesis with water–ethanol (1:1 v/v). **c** SEM image of modified HKUST-1 particle with hydroquinone at 150 °C for 16 h, showing the macroporous microparticles. **d** Macropore size distribution as measured by Hg intrusion porosimetry, with an intrusion pore volume 2.65 cm^3^ g^−1^, for the modified HKUST-1 particles. Reprinted with permission from Ref. [95]. Copyright 2014 Royal Society of Chemistry

**Fig. 12:**
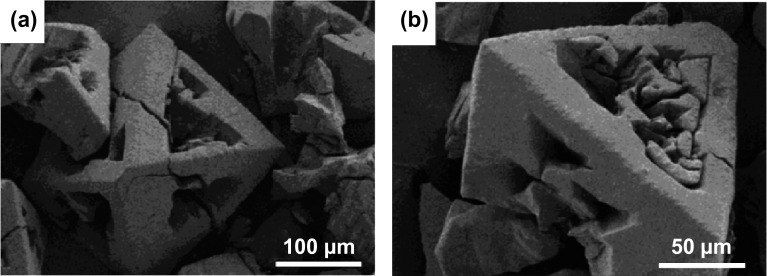
SEM images of HKUST-1 particles modified by different reagents other than hydroquinone. **a** in 50 µL boric acid aqueous solution at 150 °C for 16 h and **b** in 50 µL NaCl aqueous solution at 150 °C for 16 h, showing some large etched holes were formed from the centre of the particles. Reprinted with permission from Ref. [95]. Copyright 2014 Royal Society of Chemistry

A similar idea was developed by Ahmed et al. [95] with their report on solvothermal post-synthetic modification of microporous HKUST-1 (Fig. 11a). By etching prepared HKUST-1 in weakly acidic hydroquinone (*pK*_*a*_ = 9.85 in water) at 150 °C for 16 h, macroporous channels with pore sizes ranging from 500 nm to 10 µm (via Hg intrusion porosimetry—see Fig. 11c, d) were observed within HKUST-1 microparticles while retaining the structure of the large crystallites and high surface area. The etching process was further investigated at 120 and 180 °C for 16 h, and 150 °C for 72 h, indicating that the degree of macropore formation was dependant on reaction time and temperature. By using ICP analysis of HKUST-1 on the etching solution, it was found that Cu(II) ions were selectively removed from the framework when using a hydroquinone solution. This was also confirmed by a slight distortion in the single-crystal XRD patterns, likely caused by Cu deposits generated during the modification process. Other reagents (including H_3_BO_3_ and NaCl) were also tested in this process. Etching HKUST-1 in H_3_BO_3_ and NaCl resulted in the formation of large etched holes at the centre of the particles (Fig. 12a, b), yielding additional macropores in this MOF. The synthesised macroporous HKUST-1 crystals were then successfully used to separate styrene and ethyl benzene when packed into a high-performance liquid chromatography (HPLC) column.

**Fig. 13:**
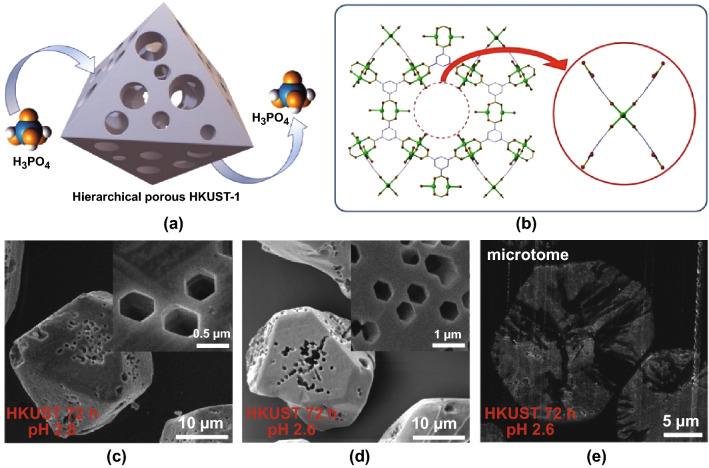
**a**, **b** Schematic representation of the etching process for HKUST-1 using phosphoric acid, showing phosphoric acid diffusing into HKUST-1 to form a hierarchical porous structure **(a)** and the disassembly of a cluster and 4 linkers (**b**). **c–e** SEM images of HKUST-1 etching in phosphoric acid using DMSO and MeOH as dilute solvents at pH 2.8 (**c**) and pH 2.6 (**d, e**), showing interconnected geometrical macropores after etching. Reprinted with permission from Ref. [96]

Prompted by the size-selective acid diffusion mechanism, a defective HKUST-1 was synthesised by Doan et al. [96] using a phosphoric acid etching agent (Fig. 13a). The mechanism of macropore formation was explained by the disassembly of one copper cluster and the paddle wheel linkers in the framework of HKUST-1 by phosphoric acid (Fig. 13b). The degradation problem of HKUST-1 in water was effectively solved by employing DMSO and methanol as dilute solvents. More interestingly, a range of interconnected highly geometrical macropores were observed with the extent of porosity being tailorable by controlling time and pH in these systems (Fig. 13c–e). It is anticipated that the macropores in these MOFs would improve the diffusion and accessibility of bulky molecules to active sites in catalysis, as was shown to be the case of defective UiO-66 which showed improved catalytic performance in Lewis acid-catalysed reactions [97].

As an extension to the idea of size-selective acid etching, another post-synthetic process, termed “synergistic etching and surface functionalisation”, was reported by Hu et al. [98]. In this study, weak bulky phenolic acids such as gallic acid and tannic acid were used to provide free protons that penetrated into the MOF crystals to form a hollow structure. Interestingly, these acids with large molecular sizes simultaneously blocked the exposed surface of ZIF-8, leading to the preservation of the parent crystalline framework of MOF in the outer shell. Yoo and Jeong [99] reported that by etching IRMOF-3 with cyanuric chloride and tetraethylamine (TEA), protons released from the modification can create a mesoporous structure or even macroscale defects if the etching process proceeds further. In this case, TEA was used as a proton scavenger to prevent the complete dissolution of IRMOF-3 in cyanuric chloride, thus preserving the MOF crystallinity.

From the above examples, it can be seen that acid etching approaches for the preparation of hierarchical macroporosity in MOFs are a comparatively facile procedure compared to templating methods, as they can be carried out as a post-synthetic step on larger samples of MOFs. Furthermore, it is possible for the defective MOFs resulting from these approaches to possess well-defined crystal structures and preserve their external morphology after acid etching, though judicious choice of the etching agent and etching conditions are essential to obtain controllable macroporosity in MOFs without the loss of crystallinity or complete dissolution of the crystalline structure. While water unstable MOFs were considered as limited candidates in some etching mechanisms, this could be solved by varying the dilute solvents employed. The nature of the interactions of various solvents with MOF frameworks therefore needs to be well understood to generate a method applicable to different MOF systems.

### Compressed or Supercritical Carbon Dioxide Method

**Fig. 14:**
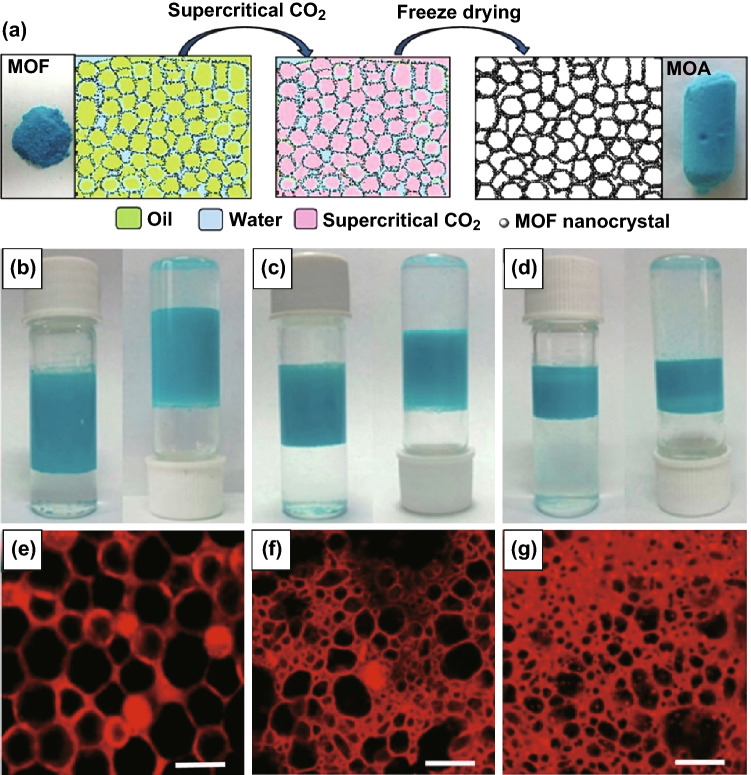
**a** Diagram illustrating the MOF-stabilised HIPE and derivation of MOA from HIPE. **b–d** Photographs of the emulsions stabilised by Cu_3_(BTC)_2_ with the initial diethyl ether volume fractions of 0.57, 0.43, and 0.29, respectively, demonstrating ability to tune pore size in HIPEs. **e**–**g** The corresponding confocal laser scanning microscopy images of the above HIPEs (HIPE-1, HIPE-2, and HIPE-3, respectively). Scale bars, 20 µm. Reprinted with permission from Zhang et al. [109]. Copyright CC BY-NC-SA 4.0

Compressed or supercritical carbon dioxide has been widely applied to a range of MOF processing steps including crystallisation [100–102], impregnation [103], dispersion [104], drying [105], and activation [106, 107]. Formation of highly porous materials will generally benefit from the use of supercritical drying (typically scCO_2_), with the low viscosity and high diffusivity of supercritical fluids being recognised as being especially vital for activation of large pores, to avoid pore collapse in MOFs during solvent removal [108]. An alternative approach for generating microporous structures in MOFs, as opposed to depositing a MOF on a porous template which has then to be removed, is to directly form microporous MOFs into an aerogel, establishing free-standing monoliths with added macroporosity. Drying with scCO_2_ has been illustrated as a key post-synthetic treatment step for synthesis of macroporous MOF/aerogel composites (MOFAC) in the absence of a polymer template. In this synthesis, a prepared MOF may be added into an emulsion to form a stable network, before drying by scCO_2_ to obtain an aerogel. Recently, a strategy to synthesise HKUST-1 MOF-stabilised high internal phase emulsions (HIPE) at the oil–water interface was described by Zhang et al. [109]. After removing the liquids with scCO_2_ (Fig. 14a), the as-assembled MOF aerogel presented a macroporous structure with an outstanding volume expansion and low density of 0.015 g cm^−3^ (Fig. 14e–g); the macropore size could be easily tuned by changing the initial oil volume fraction of the emulsion structures without the need of single-use sacrificial polymer templates (Fig. 14b-d). Similarly, Li et al. [110] reported a two-step gelation of MOF nanoparticles, in which AlBTC and AlBDC gels with different metal-to-ligand ratios and concentrations were formed at elevated temperatures before exchanging the pore liquid with liquid CO_2_ under subcritical extraction to produce meso-/macroporous “Al-MOA” aerogels. These Al-MOAs were tested for the uptake and transportation of bulky molecules, and the retention of hierarchical porosity was demonstrated by uptake of Congo red (CR) and brilliant blue R-250 (BBR-250) dyes, as studied using UV–Vis spectroscopy. It was noted that this method relies heavily on the use of subcritical CO_2_ to avoid the collapse of porous networks and hence required careful solvent removal and specialised high pressure equipment to retain the open porous structure. These MOF aerogel composites can be produced as free-standing monolithic porous solids which can be easily formed into desired shapes for numerous applications [111, 112] and hold significant advantages over powdered materials, including ease of handling, immobilisation of particulates, and improved robustness and mechanical properties. Thus, monolithic MOF aerogels may also be more practically used as catalysts and catalyst supports themselves without the restrictions in mass transport and catalyst recovery associated with nanocrystalline MOF powders.

In addition to its use in porous material activation and aerogel formation, compressed or scCO_2_ has been recently investigated as an unconventional solvent for direct MOF synthesis. By varying CO_2_ pressure in the reactor, particle sizes and porosity can be controlled, which is not possible using conventional solvothermal synthesis. These substantial benefits are reasons why using this alternative solvent in MOF synthesis can be advantageous even though a high pressure is required.

**Fig. 15:**
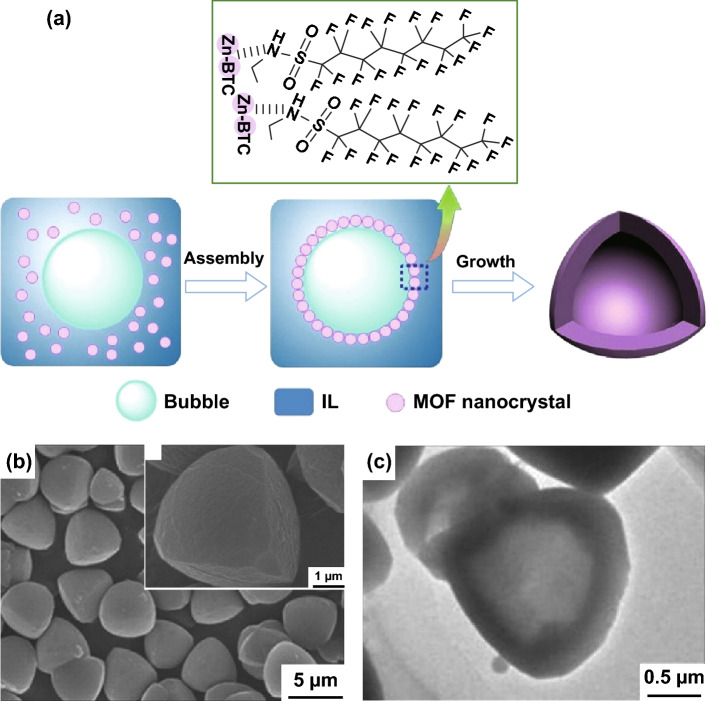
**a** Schematic illustration for the formation of hollow Zn–BTC tetrahedroids via a CO_2_–IL interfacial templating route. **b** SEM and **c** TEM images of the hollow tetrahedron-like Zn–BTC microparticles. The N-EtFOSA concentration is 2.0 wt% based on IL and the CO_2_ pressure is 6.3 MPa. Reprinted with permission from Ref. [113]. Copyright 2014 Elsevier Inc

As an early demonstration of this synthetic strategy, Peng et al. [113] described a CO_2_–ionic liquid interfacial templating route for the synthesis of hollow Zn-based MOF polyhedra. By varying the pressure of gaseous CO_2_ from 10 to 63 bar in a solution of *N*-ethyl perfluorooctylsulfonamide/*N*,*N*,*N*′,*N*′-tetramethylguanidinium trifluoroacetate (*N*-EtFOSA/TMGT), tetrahedron-like particles were formed as CO_2_ bubbles were generated (Fig. 15a). It was seen that at high pressure, the MOF tetrahedroids possessed a hollow structure with inner cores evidenced by SEM (Fig. 15b), TEM (Fig. 15c), and gas sorption. The formation of this structure was explained by the selective assembly of metal nodes and organic linkers at the bubble interfaces during the crystallisation. This MOF was then tested in the reaction of propylene oxide and CO_2_ to produce propylene carbonate, indicating an increase in conversion from 20 to 97% with the presence of the hollow structure.

**Fig. 16:**
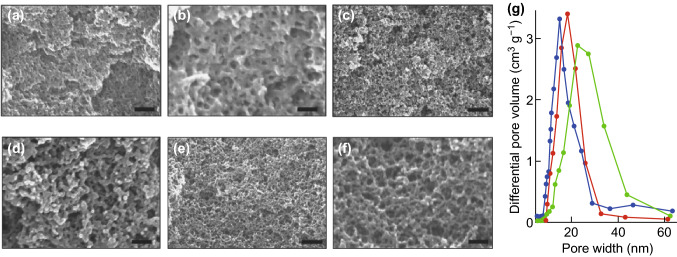
**a**–**f** SEM images of HKUST-1 synthesised in CO_2_-expanded DMF. **a**, **b** 2.0; **c**, **d** 4.5; **e**, **f** 6.6 MPa. Scale bars, 150, 50, 500, 150, 500, and 150 nm for **a**–**f**, respectively. **g** The mesopore size distribution curves for the Cu_3_(BTC)_2_ synthesised in CO_2_-expanded DMF at 2.0 MPa (blue curves), 4.5 MPa (red curves) and 6.6 MPa (green curves). Reprinted with permission from Peng et al. [114]. Copyright CC BY-NC-SA 4.0. (Color figure online)

At the same time, Peng et al. [114] also reported a method using CO_2_ to form an expanded solvent to synthesise HKUST-1 (Fig. 16a–f). It is noticeable that large mesopores (up to 30 nm) with pore wall diameters of around 10 nm could be formed within this MOF by increasing pressure to 66 bar at 30 °C for 3 h (Fig. 16g), resulting in improved performance in catalysed reactions with benzyl alcohol oxidation. The mesocellular formation in CO_2_-expanded liquids was explained by the assembly of HKUST-1 nanosized framework building blocks in expanded liquid volumes at CO_2_ pressure. While the formation of larger mesopores was favoured by higher CO_2_ pressures, this study was limited to introducing CO_2_ at 66 bar and 30 °C; the maximum pore sizes witnessed were ~ 30 nm. It can be anticipated that these mesopores need to be further enlarged in some cases to obtain macropores to more significantly enhance the transportation of reactants, intermediates, and products in heterogeneous catalysis applications.

**Fig. 17:**
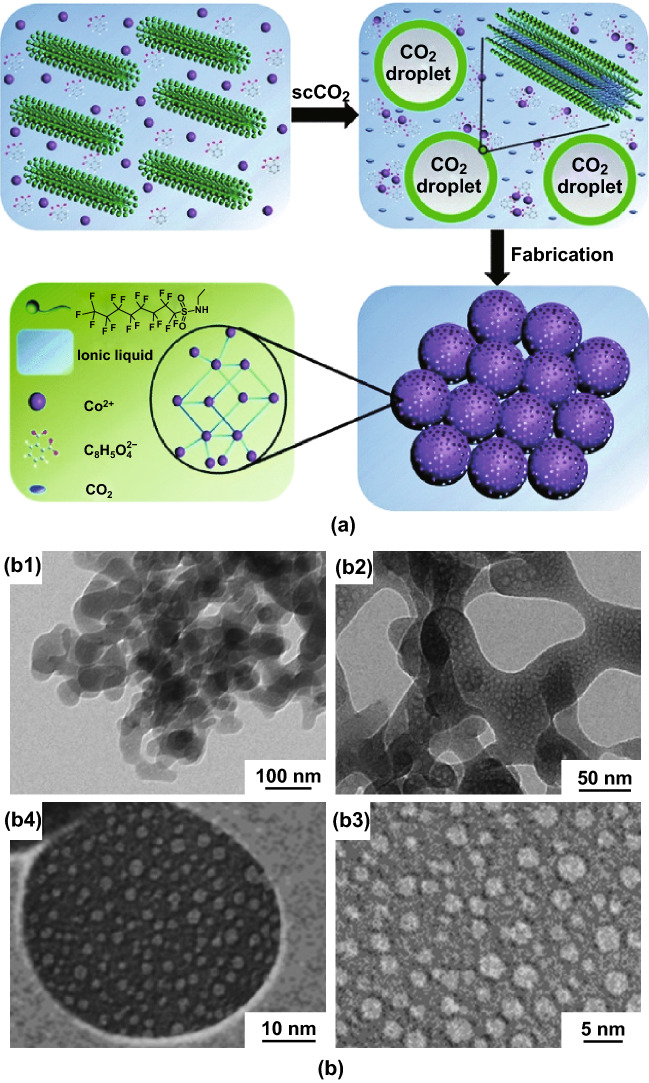
**a** Schematic illustration for the formation of the mesoporous Co-MOF in an IL/SC CO_2_/surfactant emulsion system. **b** TEM images of the Co-MOF synthesised in an IL/SC CO_2_/surfactant emulsion system at 16 MPa and 80 °C for 48 h. Reprinted with permission from Ref. [115]. Copyright 2015 Royal Society of Chemistry

Utilisation of scCO_2_ in binary solvent systems comprising an ionic liquid (IL) was also reported for the synthesis of mesoporous zinc and cobalt MOFs (Fig. 17a) [115, 116]. In these studies, scCO_2_ not only established the pattern of the MOF nanospheres but also swelled the IL micelles to form long-range ordered mesopores which could be tuned easily by changing the CO_2_ pressure. Remarkably, in the case of Co-MOF [115], a series of interconnected macropores could also be seen with a worm-like morphology (Fig. 17b). The resulting Co-MOF was shown to possess high specific capacitance values (230.5 F g^−1^ at 0.5 A g^−1^). Note that these MOF syntheses were investigated at supercritical conditions of CO_2_ (160 bar, 80 °C) and for extended reaction times (48 h) which differ from conditions chosen in Peng’s research introduced previously. The extreme reaction conditions in CO_2_ have also been employed in the synthesis of 1D, 2D, and 3D MOFs by reactive crystallisation in the absence of or using lower volumes of solvents [117–119].

**Fig. 18:**
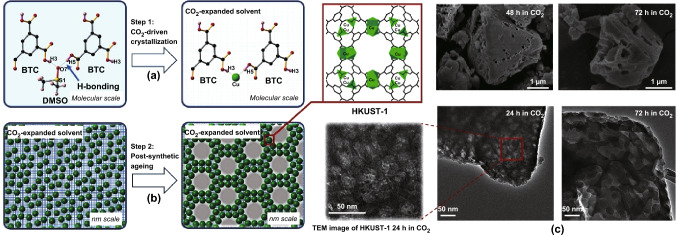
Schematic illustration for **a** HKUST-1 crystallisation and **b** meso/macropore formation in the CO_2_-expanded solvent. **c** SEM images (top) and TEM images (bottom) of HKUST-1 synthesised using scCO_2_ at 75 bar, 40 °C for more than 24 h, showing introduction of interconnected macropores. Reprinted with permission from Doan et al. [120]. Copyright CC BY-NC-SA 4.0

By tuning the conditions used in previous studies, Doan et al. [120] demonstrated the synthesis of HKUST-1 using scCO_2_ as a pressure-tuneable antisolvent to induce MOF crystallisation from the resulting expanded liquid phase (Fig. 18a). Interestingly, this methodology allowed control over nucleation and growth kinetics and also introduced a range of larger macropores into these typically microporous materials leading to so-called hierarchical porous materials (Fig. 18b). The ability to control the expansion of the solvent by control of the density of CO_2_ permits access to new structures and material morphologies not accessible by traditional methods. SEM and TEM images of the materials showed that crystallites were of the order of ~ 100 nm to microns in size, with interconnected macropores ranging from ~ 50 to 200 nm in diameter (Fig. 18c). A view cell experiment (allowing direct observation of the reactants under high pressure scCO_2_) showed that HKUST-1 could be formed within a few minutes rather than the 18 h required for the conventional synthesis [121]. In addition, due to the expansion in volume with CO_2_ pressure, the amount of the methanol antisolvent required was greatly reduced in comparison with the conventional synthesis.

The latest research demonstrates that MOFs with macropores can be synthesised using scCO_2_. These findings suggest an intelligent method to obtain an open macroporous structure without using templates. It is anticipated that this process may be applicable to other MOF systems. Note that in the case of HKUST-1 metal–ligand coordination forces between Cu^2+^ and BTC^3−^ in DMSO dominated the MOF aggregation process, resulting in a precursor solution [122]. It was found that the nucleation was only trigged when the introduction of CO_2_ and a small amount of methanol served to disrupt the H-bonding in the stock solution. Due to critical conditions of CO_2_ applied in this method (*T*_c_ and *P*_c_ of CO_2_ are 31.1 °C and 73.9 bar, respectively [123]), a high pressure reactor needed to be employed. Also, low temperature synthesis at 40 °C was found to be more favourable, which is not easy to achieve for most MOFs. Another challenge in this synthetic strategy is carrying out the experiment in in situ characterisation to understand the mechanism of the macropore formation during CO_2_ pressurisation.

### 3D Printing Method

The formation of macropores in MOFs by the acid etching and scCO_2_ methods has been shown to be beneficial for catalytic applications, but, with the exception of the MOF aerogels, most still result in MOF materials consisting of small crystallites, rather than controllable monolithic structures. As discussed in the preceding sections, a key barrier to the implementation of MOFs in industry is their lack of processability owing to their general insolubility and inherently powdered form. While a variety of methods have been attempted to transform MOF powders into appropriate monolithic forms, such as pressing powders into pellets [124], melting them to form glasses [125–127], or forming MOF coatings on monolithic supports (such as those mentioned in Sect. 2.1) [128], typically such techniques result in reduced MOF surface area.

Over the past few decades, 3D printing, also known as additive manufacture or robocasting technology, has been developed and applied successfully to fabricate monoliths with controlled 3D shapes by careful placement of material in space. Currently, printing methods are not limited to utilising inks comprised of plastics for art exhibitions and may also employ inks comprised of ceramics, metals, graphene additives, and even stimuli-responsive hydrogel composites [129] for prototyping and industrial applications [130–132]. Recently, there have been a growing number of research papers on developing tailored porous biomedical scaffolds [133], membranes [134–136], carbons [137], zeolites [138–140], aminosilicate adsorbents [141], and heterogeneous catalytic systems [142] by taking advantage of this technique.

In comparison with other processing techniques for making microporous MOFs such as solvothermal and microemulsions, 3D printing promises multiple advantages such as high reproducibility of complex geometries, low cost, scalability, and efficiency. Furthermore, with regards to MOFs specifically, the printing of a MOF composite would represent major advantages over alternative methods of monolith formation, which generally involve the deposition of MOFs onto non-flexible substrates like metals and require complicated techniques for precise MOF positioning [143, 144]. Additionally, a further benefit is the formation of a printed monolith has potential to enable additional porosity to be implemented depending on the additives employed in the ink. Hence, 3D printed monoliths not only offer an implementable contact medium for MOFs, but also enable an increased pore size distribution to be attained compared to parent MOF powders. In addition, polymers with a variety of unique attributes, such as softness, thermal and chemical stability, and optoelectrical properties, can be integrated with MOFs to make hybrids with sophisticated architectures [145]. With numerous potential applications, 3D-printed MOFs have been prepared successfully, though only recently, and therefore have been studied to only a limited extent.

**Fig. 19:**
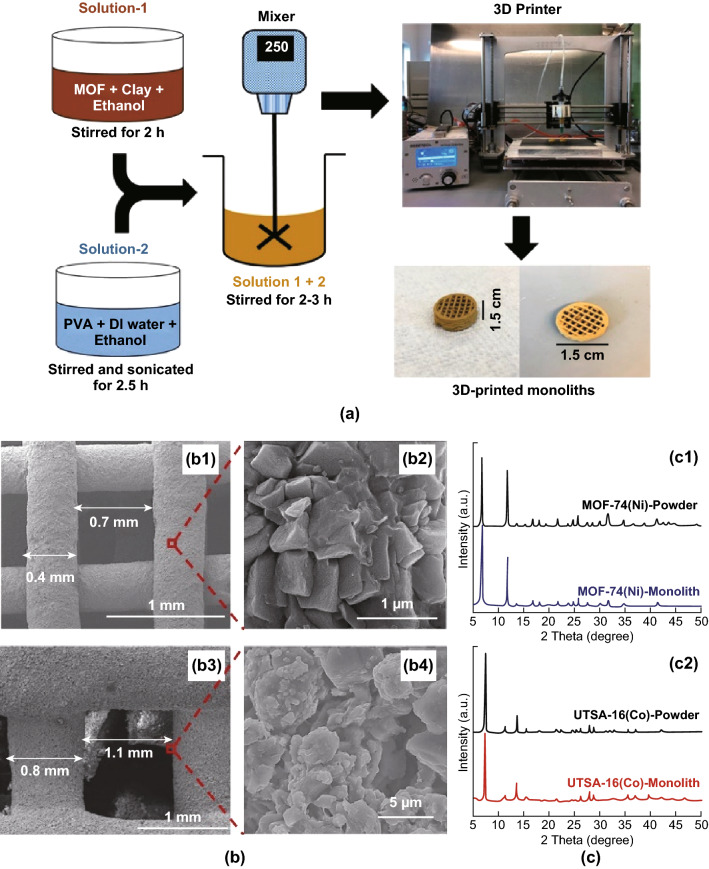
**a** Schematic of the 3D-printed MOF monolith preparation procedure. **b** SEM images of 3D-printed MOF-74 (Ni) (**b1** and **b2**) and 3D-printed UTSA-16 (Co) (**b3** and **b4**), showing uniform distribution of MOF crystals and with large voids. **c** PXRD patterns of 3D-printed MOF-74 (Ni) (**c1**) and 3D-printed UTSA-16 (Co) (**c2**) with their powder counterparts, showing crystallinity retained for both MOF-74 (Ni) and UTSA-16 (Co) MOFs after they were extruded into the monolith form. Reprinted with permission from Ref. [146]. Copyright 2017 American Chemical Society

The first report in which MOF-based inks were developed and printed to give monoliths was described by Thakka et al. [146] and employed MOF-74(Ni) and UTSA-16(Co). In this study, pre-prepared MOF powders and bentonite clay (as a binder and rheological modifier) were dissolved in ethanol to obtain a homogeneous solution. This solution was then combined with a mixture of polyvinyl alcohol (PVA) and deionised water to form an extrudable paste, before loading into a 3D-printer to create macroscopic MOF-based objects with desired shapes, as illustrated in Fig. 19a. The obtained 3D-printed MOF monoliths (at 80 and 85 wt% loadings) retained the physical properties and mechanical integrity of their powder counterparts and also displayed crystallinity akin to their parent MOF crystal structures. While physical properties were retained, the surface area of the 3D-printed MOF composites was reduced by a maximum of 38% (from 1180 to 737 m^2^ g^−1^), owing to the inclusion of bentonite clay and PVA additives. The key challenge underlined by the work of Thakka et al. was the effect of ink rheology on the resulting printed structures, the cross-sectional areas of which are displayed in Fig. 19. Differences in wall thicknesses and channel sizes were attributed to the viscosity of the prepared MOF pastes, with the less viscous UTSA-16(Co) paste resulting in the expansion of layers on deposition. Nevertheless, the printed MOF monoliths displayed CO_2_ sorption capacities similar to their MOF powder counterparts with stable performance and faster adsorption kinetics than those of the corresponding powder (by 15 min).

According to Semino et al. [147], the general method developed by Thakka et al. for the production of hierarchical MOF monoliths is likely governed by the polymer it is embedded in: firstly by the partial blocking of MOF pores by flexible polymers (giving smaller pore sizes) and secondly by the formation of voids within the polymer matrix itself. The influence of polymer characteristics on the fabrication of hierarchical MOF monoliths by 3D printing was in fact investigated by Evans et al. in 2018 [147]. In this study, high loadings of powdered ZIF-8 (up to 50 wt%) were incorporated into a matrix of either polylactic acid (PLA) or thermoplastic polyurethane, representing rigid and flexible polymer matrices, respectively, to produce a filament feedstock for 3D printing. It was revealed that the rigidity of the PLA chains limited their reorganisation around ZIF-8 crystals during extrusion, resulting in various void sizes (micro-, meso-, and macro-voids) and hence giving a hierarchical MOF monolith with preserved MOF crystallinity. Unfortunately, employment of the flexible polymer matrix resulted in almost complete pore occlusion, as concluded from the low specific surface area of the monolith (68 m^2^ g^−1^ at 50% MOF loading).

Recently, Young et al. [148] highlighted a practical solution to the problem of pore occlusion in MOF composite monoliths by 3D-printing UiO-66 in combination with a mixture of flexible acrylates, which exhibited low thermal stability (< 100 °C). As expected, the printed UiO-66 monoliths were non-porous (owing to blockage by flexible acrylate chains); however, on heating to 100 °C, the polymer matrix degraded to recover porosity and reveal UiO-66 sites. Interestingly, although the treated composite displayed physisorption behaviour characteristic of microporous UiO-66, the presence of small mesopores was also identified. This work, carried out by Young et al., promises a method to embed MOFs within 3D printed monoliths and later exposes crystals as required for production of structures with hierarchical porosity. Furthermore, UiO-66 was found to behave as a rheological modifier itself, avoiding the need for additives such as the bentonite clay employed in earlier studies.

**Fig. 20:**
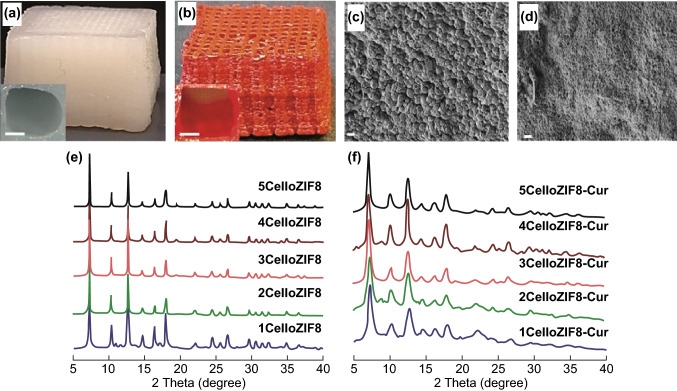
3D printed scaffolds: **a** 4CelloZIF8 and **b** 4CelloZIF8-Cur. Insets are images representing the pores, with the scale bar representing 0.5 mm. SEM images of **c** scaffold 4CelloZIF8 and **d** scaffold 4CelloZIF8-Cur. Scale bar = 1 µm. **e** PXRD patterns of CelloZIF8 hybrids using different ZIF-8 and **f** different ZIF-8 loadings while keeping Hmim:Zn to 35:1 and curcumin to 30 mg, showing that the crystallinity and the framework are maintained with different curcumin and Zn loadings. Reprinted with permission from Ref. [149]. Copyright 2019 Wiley-VCH Verlag GmbH & Co. KGaA, Weinheim

While the most commonly employed methods to prepare printable MOF inks combine synthesised MOFs and additives (such as binders and plasticisers) in ethanol, a novel one-pot room temperature approach using water as a solvent was carried out by Sultan et al. [149]. The printable ink was formulated by in situ growth of ZIF-8 and MIL-100(Fe) MOFs onto anionic 2,2,6,6-tetramethylpiperidine-1-oxylradical-mediated oxidised cellulose nanofibers (TOCNFs) prior to combination with a sodium alginate binder and calcium chloride crosslinker. Introducing MOFs into cellulose resulted in increased pore volume and surface area of the composite material compared to unmodified TOCNF. In contrast, the cellulose (with high aspect ratio, negative zeta potential, and good mechanical performance) provided benefits to MOFs by offering high printability and versatility of the hybrid inks. The printed scaffolds (Fig. 20a–d) possessed a large pore size (1 mm), useful for drug delivery applications, as the large pores would aid in infiltration of biological fluids into the support. In addition, the 3D-printed composite was found to be stimuli responsive (pH dependent) regarding the release of curcumin and methylene blue, indicating that the printed MOF monoliths produced using a simple and inexpensive printer could potentially be used in biomedical applications.

Recent developments in the fabrication of 3D-printed MOFs have been successful in both providing a monolith suited for practical applications and adding hierarchical porosity into MOF-based materials. Although 3D printing of MOF monoliths has predominantly required high processing temperatures (up to 230 °C) or ultraviolet curing [150, 151], the work described by Sultan et al. demonstrated the feasibility of room temperature synthesis. However, it should be noted that this technique may not be suited to a variety of MOFs which are unstable in air, since the layer-by-layer printing process may necessitate air exposure for extended periods (15–120 h). For example, Kreider et al. [152] observed the degradation of water-sensitive MOF-5 during blending of the printable ink with the MOF powder and attributed this to ambient humidity. Interestingly though, irrespective of the observed MOF degradation, the resulting monolith was capable of adsorbing more H_2_ than the pure polymer counterpart, demonstrating the viability for H_2_ storage using 3D printed MOFs even in humid conditions.

Overall, the ability to tailor both the MOF and binding matrix could render extensive applications viable, from providing photonic platforms to biomedical testing and catalysis. While the precise placement of materials in 3D printing renders more complex macroscale structures achievable, it should be noted that fine control on the micron–nanoscale could be challenging in terms of accurately controlling the size of macropores which form inherently during ink deposition. However, as 3D printing methods become more established, it is anticipated that finer control over macropore sizes will be achievable. Finally, since significant research attention has taken steps towards employing MOFs in biomolecule encapsulation for controlled drug delivery and in biological applications, where macroporosity would be beneficial for facilitating mass transfer of biological fluids, it may be useful to consider the biodegradability of MOFs in low pH environments [153, 154]. To this end, future work could explore the development of ink matrices which could protect embedded MOFs from such degradation.

## Conclusions

In this review, a range of fabrication methods for creating hierarchical MOF pore structures with additional macropores were discussed. Introduction of macroporosity has been demonstrated to lead to improvements in mass transfer and catalytic activity, and reductions in pressure drop over traditional powdered or nanocrystalline materials. These macropores can also improve molecular accessibility of reagents to the microporous cavities, which accommodate important functional groups or active sites, enhancing the catalytic performance of the materials. The advantages of macropores in catalytic applications have been well demonstrated for zeolites and metal oxides. For MOFs as microporous crystallites (which are purposely designed to possess high surface areas), introducing additional meso- and macropores to form hierarchical structures without compromising the micropores and the crystallinity, which give them high surface areas, is becoming the subject of much research. While mesopores can be created via numerous methods such as ligand exchange and use of surfactants, further expanding these to the macropore regime remains a challenge due to the ligand size limitation and the post-synthetic activation methods needed to obtain accessible pores.

As shown here, the main techniques reported for fabrication of macroporous materials can be described using four general approaches: use of structural templating, defect formation, use of CO_2,_ and 3D printing. Templating stands out as the most popular route towards obtaining macroporous MOFs with numerous studies reported so far, due to the ease of structural control and the ease with which this method can be adapted for use with a range of MOFs. Deposition of MOFs onto hard macroporous templates and the formation of MOF composites via direct synthesis onto a porous template often do not require significant deviation from the synthetic conditions identified for the MOF itself and may offer an additional advantage in improving mechanical and thermochemical stability and providing immobilisation of nanocrystalline MOF materials. This may improve the outlook for MOFs to be used in industrial catalysis, for example, in alkylation and fluid catalytic cracking in oil refining processes involving bulky hydrocarbons, where harsh conditions require higher catalyst stability. In addition, the use of a hard template means that the active MOF material may only constitute a small proportion of the overall composite by weight, which may be advantageous in the case of more expensive MOF materials. In post-treatment synthetic methods, pre-formed MOFs can be immobilised on macroporous templates such as foams and sponges using dip-coating methods to form the composites. The stability of the MOFs used in this method remains a major challenge due to a long exposure time to the high humidity conditions during the immobilisation process, though this may be addressed through careful choice of solvents. In addition, in these macroporous composite structures, the MOF materials must be suitably bonded to the surface (e.g. via functionalisation of the template surface) to avoid being washed off over time.

If, rather than rigid composites, flexible composites or pure macroporous MOF structures are required, soft macroporous templates such as polymers, emulsions, or gels can be used. Polymer templates can allow for more pliable macroporous structures, while the use of emulsions or gels may allow for easier removal after templating. Use of sacrificial soft templates can result in macroporous stand-alone aerogel monoliths which can lead to easier and safer materials handling for particular applications; however, careful template removal is required to avoid incomplete removal (which could lead to loss of porosity) or collapse of pore networks.

Defect formation can be implemented via direct synthesis using linker modulation techniques or post-synthetic acid etching of prepared MOFs in low pH environments. The former has been widely reported for synthesis of mesoporous MOFs with defects as active adsorption and reaction sites for enhanced catalytic activity and gas storage, with linker modulation now being explored for introduction of larger macroporous voids. Defect formation via acid etching can overcome the limitation of the need for extended ligands and can potentially create macropores via size-selective diffusion processes. In fact, various etching agents such as hydroquinone, boric acid, and phosphoric acid have been studied so far to synthesise some impressive macroporous MOF crystal structures. While this method remains challenging due to the instability of most MOFs under acidic conditions, there have been promising recent developments, such as the discovery that careful selection of solvents can allow large geometrical pores within HKUST to be achieved by acid etching and that the use of synergistic etching to protect surfaces can be used to control etching. These examples indicate that with further investigation of the synthetic conditions and further understanding of the etching mechanisms, this simple approach to the formation of such macropores in MOF crystallites may be more widely applied.

Utilising supercritical or compressed CO_2_ in MOF preparation can address some key challenges noted in previous methods including pore collapse during solvent removal steps when using soft structural templating. In fact, this method is well known in MOF synthesis and post-synthetic treatment and has showed some promising results in creating open macroporous aerogel structures. Further adapting the synthetic conditions (e.g. longer reaction times and higher pressures), the use of scCO_2_ routes was shown to be able to form additional macropores in the direct synthesis of MOFs. In HKUST-1 synthesis, this method was also demonstrated to quickly trigger the reaction with a lowered amount of solvent, and as an added advantage, due to the insolubility of scCO_2_ in stock solutions, the multiple purification steps required in conventional synthetic methods could be curtailed. Particle sizes and porosity of this MOF can be tuneable via tight control over etching time. This route offers substantial benefits in HKUST-1 preparation compared to conventional solvothermal method. However, the mechanisms of the macroporous HKUST-1 formation need to be fully understood, before generalising this strategy to other MOF systems.

Finally, 3D printing is a very new fabrication technique which shows promise for generalised use in MOF preparation, allowing a very high MOF loading to be incorporated into porous printed structures (~ 85 wt%). The high reproducibility, fabrication of complex geometries, and controlled pore structures of these MOFs have potential to open up new applications for MOF materials in biomedical fields. This method, however, relies on the ability to incorporate MOFs and MOF precursors into inks capable of being printed into stable structures without compromising on the nanoscale structure of the MOF. Enabling higher resolution (sub-mm) printing is also an area for future development, to enable greater control over the macrostructure, for example in biomedical applications such as tailored drug delivery or the size-selective diffusion of proteins to oxidation sites within MOFs facilitates biocatalysis.

As shown by many of the studies mentioned in this review, macroporous MOF structures can show enhanced performance in adsorptive and catalytic applications due to the improved mass transfer and molecular accessibility. It can be predicted that more research will be carried out to produce more useful composites of functional MOFs coordinated with macroporous substrates using reproducible and sustainable synthetic approaches. The range of fabrication approaches presented here indicates the vast variety of different macroporous structures that can be achieved using functional MOF materials. The ability to include structural macroporosity should help to accelerate the practical application of MOFs in such varied fields as large molecule adsorption and separation, water purification, bulky drug delivery, and heterogeneous catalysis. Further development of this toolbox will surely give material scientists greater flexibility in tailoring the porosity and structure of their MOF materials towards particular functions.
